# Risk Factors and Associated Outcomes of Virulence Genes eae, entB, and pipD Carriage in Escherichia coli, Klebsiella pneumoniae, and Salmonella spp. From HIV-1 and HIV-Negative Gastroenteritis Patients in the Dschang Regional Hospital Annex

**DOI:** 10.7759/cureus.42329

**Published:** 2023-07-23

**Authors:** Ngangoum G Djomgoue, Leinyuy J Fonbah, Ali I Mbulli, Karimo Ousenu, Tume C Bonglavnyuy

**Affiliations:** 1 Research Unit of Microbiology and Antimicrobial Substances, Department of Biochemistry, University of Dschang, Dschang, CMR

**Keywords:** hiv-positive patients, salmonella spp, k. pneumoniae, e. coli, severity of symptoms, antibiotic resistance, virulence genes, gastroenteritis

## Abstract

Background

*Enterobacteriaceae* is one of the main families of gram-negative bacilli responsible for serious infections in humans. The severity of infection by these bacteria is a product of many factors, including virulence properties and antimicrobial resistance. This severity may be further intensified if there is an association between these factors and a depressed immune system, such as in HIV patients. This study aimed to determine the distribution of representative virulence genes among key *Enterobacteriaceae* isolates from HIV-1 and non-HIV gastroenteritis patients and the relationship between carrying these virulence genes and antimicrobial susceptibility, seropositive status, and severity of symptoms associated with *Enterobacteriaceae *infections in Dschang Regional Hospital Annex.

Methodology

A total of 200 gastroenteritis patients (100 HIV-1 and 100 non-HIV patients) were selected and evaluated for symptoms associated with gastroenteritis. Stool samples were obtained and cultured, from which *Escherichia coli*, *Klebsiella pneumoniae,* and *Salmonella* spp. isolates were obtained. Antibiotic susceptibility tests were performed on the isolates by agar disc diffusion using commonly used antibiotics. These isolates were tested for the possession of virulence genes by polymerase chain reaction (PCR); *eae* for *E. coli*, *entB* for *K. pneumoniae,* and *pipD* for *Salmonella *spp. Correlation tests and risk assessments were performed between the presence of virulence genes, antibiotic resistance, and specific symptoms.

Results

The isolates obtained from HIV-positive and HIV-negative patients were, respectively, 61 against 62 for *E. coli*, 10 against 21 for *K. pneumoniae,* and 11 against 15 for *Salmonella* spp.These organisms showed the highest resistance to amoxicillin and clavulanic acid, while the least resistance was observed against ofloxacin, gentamicin, and amikacin in both groups of patients. The virulence genes showed a generally higher occurrence in isolates from HIV-negative patients than HIV-positive patients, with the *eae *gene 5/61 (8.20%) against 12/62 (19.35%), the *entB gene* 4/10 (40.00%) against 14/21 (66.66%), and the *pipD* gene 5/11 (45.45%) against 7/15 (46.46%) in HIV-positive and negative patients, respectively. There was a significant correlation between *eae *gene carriage and resistance against imipenem (p = 0.047), gentamycin (p = 0.047), and doxycycline (p = 0.029); *entB* gene carriage and resistance toward levofloxacin (p = 0.017) in *K. pneumoniae*; and *pipD* gene carriage and resistance against levofloxacin (p = 0.039), imipenem (p = 0.041), and doxycycline (p = 0.042). The carriage of the virulence genes was seen to be a stronger risk only for the resistance of *K. pneumoniae* to ceftriaxone (odds ratio (OR) = 2.286) and gentamycin (OR = 3.000), and *Salmonella *spp. against imipenem (OR = 2.750) and doxycycline (OR = 2.118). The development of severe symptoms correlated significantly with virulence gene carriage in isolates, mainly in HIV-positive patients with *eae *(p = 0.017) and *pipD *(p = 0.025), with a strong risk association with the *pipD* gene (OR = 2.665).

Conclusions

Antibiotic resistance was associated with virulence gene carriage, indicating that virulence and antibiotic resistance can associate their effects and contribute to poor outcomes in the treatment of bacterial diseases in HIV patients. The possession of virulence genes increased the severity of symptoms associated with gastroenteritis in HIV-positive patients.

## Introduction

Gastroenteritis is one of the most common infectious diseases and a major cause of mortality among humans in low-income and middle-income countries [1]. Its development is greatly influenced by a compromised immune system, recent exposure to antibiotics, and the possession of virulence genes [1,2]. Most gastroenteric infections have a bacterial origin, with *Enterobacteriaceae* being the most frequent cause. In immunocompromised patients, opportunistic infections due to these bacteria have varied severity due to the weakening of the immune system leading to increased vulnerability to opportunistic infections as well the possession of virulence factors [3]. The acquisition of virulence genes can increase the pathogenicity of microorganisms and the severity of infection leading to a wide range of intestinal and extraintestinal infections including diarrhea, urinary tract infections, bacteremia, and meningitis with consequent therapy failure [4]. *Escherichia coli* pathotypes, for example, intestinal pathogenic *E. coli* (IntEC) possess various types of virulence factors such as adhesins, toxin production, hemolysins, proteases, cell surface hydrophobicity, and colicins allowing them to express their pathogenicity [5]. Adhesins increase the expression of bacterial toxins and iron acquisition and eschew the host defense mechanisms. One of the most prevalent adhesin genes is the *eae* (intimin) virulence gene [6]. For *Klebsiella pneumoniae*, the different virulence factors responsible for their pathogenicity are capsule and hypermucoviscosity, lipopolysaccharides, adhesin, iron acquisition systems, fimbriae, and biofilm formation. These genes include *enterobactin* (*entB*), *Fimbrial adhesin* (*FimH*), *regulator of mucoid phenotype A* (*rmpA*), and the *mucoviscosity-associated gene A *(magA) [7]. The *entB *is one of the most common siderophores detected in *K. pneumoniae*. It codes for iron-chelating agents siderophores with higher affinity to extracellular ferric ions and induces biofilm development and maturation [8]. The pathogenicity of *Salmonella *is mediated by numerous virulence genes comprising *invA*, *fimA*, *stn*, *spvR*, *spvC*, *spiC*, and *pipD* [9]. The *pipD *gene found in SPI-5 is a type III secreted effector, a group of virulence factors in gram-negative pathogens that modulate the infection process by enabling them to bypass the extracellular milieu and inject bacterial effector proteins directly into the host cell cytoplasm [10,11]. Understanding the link between the variation of symptoms of gastroenteritis in immunocompetent and immunosuppressed patients, the presence of virulence genes, and the distribution of antimicrobial resistance in strains of *Enterobacteriaceae *implicated in the infections will also allow for more effective prevention and management strategies. Thus, this study aimed to demonstrate the link between symptoms of gastroenteritis, the distribution of the virulence gene, and antibiotic resistance in Enterobacteriaceae isolates implicated in the disease obtained from HIV-1 gastroenteritis patients in the Dschang Regional Hospital Annex.

## Materials and methods

Study design and location

This cross-sectional study was conducted in the Dschang Regional Hospital Annex and the Laboratory of Antimicrobial Substances (RUMAS) at the University of Dschang, Menoua Division, in the West Region of Cameroon.

The West Region is the smallest of Cameroon’s 10 regions in area, yet it has the second highest population density, with a population of 1,865,394 (2013) and a density of 142.9 inhabitants per km^2^ as of 2017 [12]. It has eight divisions, namely, Bamboutos, Upper-Nkam, Upper-Plateau, Koung-Khi, Menoua, Mifi, Nde, and Noun. The Menoua Division has a surface area of 1,380 km^2^ and is divided into six sub-divisions: Dschang, Santchou, Nkong-Ni, Penka Michel, Fokoue, and Fongo-Tongo. The division has an average rainfall of 1,717.7 mm and temperatures ranging from 13.6°C to 25.35°C. Overall, 80% of inhabitants practice farming [13]. This study was conducted from March 2019 to September 2022. Field work and sampling, laboratory isolation and characterization, and antibiotic susceptibility testing were done from March 2019 to September 2021. From February 2022 to September 2022, the molecular analysis of virulence genes was performed.

Selection criteria for participants

Eligible patients included HIV-infected or non-infected patients showing one or all of the following symptoms of gastroenteritis: diarrhea, abdominal pain, nausea, fatigue, vomiting, and headache. Patients who provided consent were enrolled in the study. Demographic and clinical data were collected from participants using a structured questionnaire. Patients on antibiotics for two weeks or more before the study, those who refused to give their informed consent, and HIV/AIDS patients who were not on antiretroviral drugs were excluded.

Sample collection and culture

Approximately 3 to 4 g of fresh stool was collected using a sterile, tight-capped, wide-mouthed stool sampling bottle. The stool samples were examined for macroscopic characteristics. The stool collected was dissolved in 5 mL of physiological water, seeded on MacConkey agar and eosin methylene blue (EMB) agar by plate streaking, and incubated at 37°C for 48 hours. After incubation, isolated colonies on the EMB and MacConkey agar were identified based on colony characteristics. These isolated colonies were picked and dissolved in a conservation medium, a mixture of glycerol and Muller-Hilton broth at one part to three parts, and stored at 20°C [14].

Identification of isolates

Isolates were identified first based on their colony characteristics on the culture media. Further identification included Gram staining and a battery of biochemical tests, including urea and indole tests, catalase, oxidase, motility, and Kligler iron agar (KIA) tests. The identities of selected isolates were confirmed using the Api 20 E Gallery (BioMérieux).

Antibiotic susceptibility testing

Antibiotic susceptibility tests (ASTs) were performed on the identified isolates using the Kirby Bauer disk diffusion method on Mueller-Hinton agar (MHA) during 24 hours of incubation according to the Clinical and Laboratory Standards Institute guidelines. The antibiotics tested were chosen from families formally or presently used against *Enterobacteriaceae *infections, including amoxicillin + clavulanic acid (AMC), ceftriaxone (CRO), cefotaxime (CTX), cefixime (CFM), imipenem (IMP), gentamicin (CN), ciprofloxacin (CIP), levofloxacin (LEV), ofloxacin (OFX), amikacin (AK), tobramycin (TOB), minocycline (MI), and doxycycline (DO) [15].

Amplification of virulence genes

DNA for the detection of virulence genes was extracted from fresh colonies by heat shock. A loop-full of a fresh colony was dissolved in 200 μL of Tris-ethylenediaminetetraacetic acid (EDTA) 1× buﬀer (TrisCl 0.1 M and EDTA 0.01 M diluted 1/10). The solution was vortexed for five seconds and heated in a water bath at 95°C for 25 minutes. The heated solution was centrifuged at 13,000 rpm for three minutes and the supernatant containing DNA was extracted and used for polymerase chain reaction (PCR). The targeted virulence genes, chosen from the literature and known to be highly associated with virulence in isolated pathogens, were amplified by PCR using specific primers [11,16,17]. The PCR reaction mixture was prepared in 15 μL reaction volumes containing 1 µL of each 10 μmol^−1^ forward and reverse primers, 1.5 μL of T10×, 1 μL of MgCl_2_, 0.3 μL of dNTP, 7.14 µL pyrogen-free water and 0.06 μL of Taq polymerase, and 3 μL of µL DNA template [14]. The primers used and the PCR conditions are summarized in Table 1.

**Table 1:** Primers used and PCR conditions. The reaction conditions (temperature/duration) are given in the following order: initial denaturation, denaturation, annealing, initial elongation, and final elongation. All reactions were run for 35 cycles. F = forward, the forward primer copying the template at the 3’-5’ direction; R = reverse, the reverse primer copying the template in the 5’-3’ direction; bp = base pairs; PCR = polymerase chain reaction

Name of the gene	Primer sequence	Amplicon size	PCR condition (35 cycles)	Reference
Eae	F: TCAATGCAGTTCCGTTATCAGTT R:GTAAAGTCCGTTACCCCAACCTG	482 bp	95°C for 5 minutes, 95°C for 30 seconds, 55°C for 45 seconds,72°C for 1 minute, 72°C for 5 minutes	[16]
entB	F: ATTTCCTCAACTTCCTGGGG R: AGCATCGGTGGCGGTGGTCA	371 bp	94°C for 5 minutes, 94°C for 30 seconds, 57°C for 45 seconds, 68°C for 1 minute, 68°C for 5 minutes	[17]
pipD	F: CGGCGATTCATGACTTTGAT R: CGTTATCATTCGGATCGTAA	350 bp	95°C for 5 minutes, 95°C for 30 seconds , 58°C for 45 seconds , 72°C for 1 minute, 72°C for 5 minutes	[11]

Data analysis

This research generated data on antibiotic resistance and the distribution of the virulence genes associated with bacterial gastroenteritis linked to *Enterobacteriaceae *in HIV-positive and HIV-negative patients. Qualitative data was in the form of frequency distribution tables. The chi-square test and Fisher’s exact test were used for categorical data. Risk estimates and Spearman’s correlation tests were applied to estimate the relationship between the possession of virulence genes and gastroenteritis and between the virulence gene and antibiotic resistance. A p-value ≤0.05 was considered statistically significant. Given that all patients selected showed symptoms of gastroenteritis, the severity of symptoms for the correlation with the occurrence of virulence genes in isolates was interpreted as showing three or more of the symptoms evaluated.

## Results

Occurrence of *E. coli*, *K. pneumoniae*, and *Salmonella* spp. in patients

A total of 400 patients were recruited, 200 HIV-positive and 200 HIV-negative patients, from whom stool samples were collected. Among the *Enterobacteriaceae *isolated from the stool samples, the occurrence of *E. coli*, *K. pneumoniae*, and *Salmonella* spp. is represented in Table 2.

**Table 2:** Prevalence of Enterobacteriaceae in HIV-positive patients and non-HIV participants. N = total number of patients; n = number of isolates of a given species isolated

Organisms	N	Prevalence n (%)	
		HIV-positive	HIV-negative
E. coli	200	61 (30.50)	62 (31.00)
K. pneumoniae		10 (05.00)	21 (10.50)
Salmonella spp.		11 (05.50)	15 (7.50)

*E. coli* had the highest prevalence in both HIV-positive and negative patients. *K. pneumoniae* and *Salmonella* spp. were the least common germ isolates in HIV-positive patients.

Antibiotic resistance profiles of *E. coli*, *K. pneumoniae*, and *Salmonella* spp. isolates

AST on *E. coli*, *K. pneumoniae*, and *Salmonella *spp. isolates revealed levels of resistance against the antibiotics, as shown in Table 3.

**Table 3:** Prevalence of antibiotic resistance of the Enterobacteriaceae isolates. N is the total number of isolates of each species isolated and tested, and the number of resistant isolates against a given antibiotic is expressed over the N total isolates as a percentage. AMC = amoxicillin/clavulanic acid; IPM = imipenem; CTX = cefotaxime; CRO = ceftriaxone; LEV = levofloxacin; CIP = ciprofloxacin; OFX = ofloxacin; CN = gentamycin; AK = amikacin; TOB = tobramycin; MI = minocycline

Organisms	N		AMC		IMP		CTX		CRO		LEV		CIP	
	HIV+	HIV-	HIV+	HIV-	HIV+	HIV-	HIV+	HIV-	HIV+	HIV-	HIV+	HIV-	HIV+	HIV-
E. coli	61	62	39 (63.93)	33 (53.22)	50 (31.96)	15 (24.19)	16 (26.22)	37 (59.67)	10 (17.54)	8 (13.33)	4 (6 .55)	7 (11.29)	3 (4.91)	9 (14.51)
Klebsiella	10	21	9 (90.00)	21 (100.0)	0 (0.00)	11 (52.38)	5 (50.00)	14 (66.66)	6 (60.00)	2 (9.52)	1 (10.00)	3 (14.28)	0 (0.00)	4 (19.04)
Salmonella	11	15	7 (63.63)	11 (73.73)	4 (36.36)	0 (0.00)	3 (27.27)	3 (20.00)	4 (36.36)	2 (13.33)	4 (36.36)	0 (0.00)	4 (36.36)	1 (6.66)
Organisms	N		OFX		CN		AK		TOB		DO		MI	
	HIV+	HIV-	HIV+	HIV-	HIV+	HIV-	HIV+	HIV-	HIV+	HIV-	HIV+	HIV-	HIV+	HIV-
E. coli	61	62	0 (0.00)	5 (8.06)	0 (0.00)	2 (3.22)	1 (1.63)	2 (3.22)	0 (0.00)	7 (11.29)	13 (28.26)	15 (26.31)	20 (37.73)	17 (30.35)
Klebsiella	10	21	0 (0.00)	0 (0.00)	0 (0.00)	0 (0.00)	0 (0.00)	0 (0.00)	5 (50.00)	6 (28.57)	5 (50.00)	3 (14.28)	7 (70.00)	8 (38.09)
Salmonella	11	15	0 (0.00)	0 (0.00)	0 (0.00)	0 (0.00)	0 (0.00)	0 (0.00)	1 (9.09)	0 (0.00)	4 (36.36)	2 (13.33)	6 (54.54)	3 (20.00)

The antibiotic susceptibility patterns of 123 *E. coli*, 31 *K. pneumoniae*, and 26 *Salmonella *spp. isolates were tested against a panel of 12 antibiotics. *E. coli* (63 .93% versus 53.22%), *K. pneumoniae* (90.00% versus 100.00%), and *Salmonella *spp. (73.73% versus 63.63%) showed higher resistance against AMC both in HIV-positive and negative patients. *E. coli* was also highly resistant to IPM (31.96%) in HIV-positive patients, while *K. pneumoniae* showed higher resistance to CTX (66.66%) and to CRO (60.00%) in HIV-positive patients. The highest resistance against *Salmonella* spp. was observed against MI (54.54%).

Detection of virulence genes in *E. coli*, *K. pneumoniae*, and *Salmonella *spp. isolates

The virulence genes *eae*, *entB*, and *pipD *were amplified with occurrences shown in Table 4.

**Table 4:** Frequency of virulence genes amplified from E. coli, K. pneumoniae, and Salmonella spp. N = number of isolates tested per status; n = number of isolates carrying the virulence gene

		Number of isolates tested per status, N		Frequency of germs positive to the gene, n (%)	
Organisms	Genes	HIV+	HIV-	HIV+	HIV-
E. coli	Eae	61	62	5 (8.20)	12 (19.35)
K. pneumoniae	EntB	10	21	4 (40.00)	14 (66.66)
*Salmonella *spp.	PipD	11	15	5 (44.45)	7 (46.66)

These genes showed a relatively higher occurrence in HIV-negative patients than in HIV-positive patients.

Association of the occurrence of virulence genes with antibiotic resistance

The evaluation of the correlation of the carriage of the virulence genes *eae*, *entB*, and *pipD *with antibiotic resistance in isolates from HIV-positive patients is presented in Table 5.

**Table 5:** Correlation of virulence genes and antibiotic resistance in HIV-positive patients. LI = lower interval; UI = upper interval. The p-value is given at 95% CI and significant at ≤0.05. AMC = amoxicillin/clavulanic acid; IPM = imipenem; CRO = ceftriaxone; CTX = cefotaxime; LEV = levofloxacin; CIP = ciprofloxacin; OFX = ofloxacin; CN = gentamycin; AK = amikacin; TOB = tobramycin; MI = minocycline

	*eae* in *E. coli*		*entB* in *K. pneumoniae*		*pipD* in* Salmonella* spp.	
Antibiotic	Odds ratio (LI–UI)	P-value	Odds ratio (LI–UI)	P-value	Odds ratio (LI–UI)	P-value
AMC	0.762 (0.643–0.902)	0.154	0.914 (0.659–1.269)	0.551	1.086 (0.751–1.580)	0.679
IMP	2.522 (0.328–0.831)	0.047	0.867 (0.255–2.946)	0.827	2.750 (0.967–7.817)	0.041
CRO	0.615 (0.343–1.100)	0.185	1.333 (0.552–3.220)	0.560	0.851 (0.389–1.890)	0.719
CTX	1.384 (0.405–4.726)	0.589	2.286 (0.963–5.427)	0.080	1.630 (0.486–5.467)	0.450
CIP	0.721 (0.188–2.764)	0.652	1.300 (1.149–11.358)	0.827	0.778 (0.549–1.103)	0.110
OFX	0.553 (0.148–2.058)	0.412	3.714 (0.404–34.118)	0.234	1.833 (0.386–8.701)	0.463
LEV	1.195 (0.172–8.283)	0.859	0.667 (0.379–1.174)	0.017	0.667 (0.420–1.058)	0.039
CN	0.244 (0.092–0.651)	0.007	3.000 (0.642–14.023)	0.163	1.630 (0.486–5.467)	0.450
AK	0.714 (0.204–2.504)	0.623	1.714 (0.514–5.720)	0.415	0.917 (0.273–3.075)	0.895
TOB	0.645 (0.167–2.484)	0.548	1.625 (0.719–3.673)	0.343	0.292 (0.39–2.206)	0.211
DO	0.479 (0.295–0.775)	0.029	0.667 (0.192–2.320)	0.518	2.118 (0.980–4.883)	0.042
MI	0.634 (0.503–0.800)	0.075	1.120 (0.438–2.285)	0.563	1.571 (1.005–2.456)	0.078

There was a significant correlation between the *eae* gene carriage in *E. coli* and antibiotic resistance against IMP (p = 0.047), CN (p = 0.047), and DO (p = 0.029); the entB gene carriage and resistance toward LEV (p = 0.017) in *K. pneumoniae*; and the pipD gene carriage and resistance against LEV (p = 0.039), IMP (p = 0.041), and DO (p = 0.042). The carriage of the virulence genes was seen to be a stronger risk only for the resistance of *K. pneumoniae* to CRO (odds ratio (OR) = 2.286) and CN (OR = 3.000), and *Salmonella *spp. against IMP (OR = 2.750) and DO (OR = 2.118).

The evaluation of the correlation of the carriage of the virulence genes *eae*, *entB*, and *pipD *with antibiotic resistance in isolates from HIV-negative patients is presented in Table 6.

**Table 6:** Correlation of virulence genes and antibiotic resistance in HIV-negative patients. LI = lower interval; UI = upper interval. The p-value is given at 95% CI and significant at ≤0.05. AMC = amoxicillin/clavulanic acid; IPM = imipenem; CRO = ceftriaxone; CTX = cefotaxime; LEV = levofloxacin; CIP = ciprofloxacin; OFX = ofloxacin; CN = gentamycin; AK = amikacin; TOB = tobramycin; MI = minocycline

	*eae* in *E. coli*		*entB* in *K. pneumoniae*		*pipD* in *Salmonella* spp.	
Antibiotic	Odds ratio (LI–UI)	P-value	Odds ratio (LI–UI)	P-value	Odds ratio (LI–UI)	P-value
AMC	0.632 (0.448–0.890)	0.116	1.000 (0.804–1.244)	0.162	1.259 (0.503–3.151)	0.679
IMP	0.500 (0.2530–1.000)	0.119	0.857 (0.353–2.079)	0.741	2.182 (0.809–5.885)	0.086
CRO	0.667 (0.481–0.924)	0.146	1.833 (0.853–3.940)	0.108	1.061 (0.470–2.398)	0.895
CTX	0.556 (0.282–1.094)	0.174	1.737 (0.814–3.705)	0.142	0.833 (0.374–1.855)	0.673
CIP	1.765 (0.273–11.421)	0.541	1.207 (0.357–6.585)	0.162	0.389 (0.218–0.694)	0.110
OFX	1.250 (0.197–7.921)	0.819	1.500 (0.282–7.987)	0.642	0.667 (0.259–1.718)	0.463
LEV	1.250 (0.185–8.444)	0.824	3.230 (0.342–5.327)	0.303	0.353 (0.185–0.672)	0.039
CN	1.250 (0.711–1.886)	0.036	3.000 (0.691–5.029)	0.118	1.436 (0.551–3.740)	0.450
AK	0.842 (0.125–5.674)	0.869	1.750 (0.612–5.006)	0.296	0.942 (0.417–2.130)	0.895
TOB	0.263 (0.048–1.432)	0.126	0.875 (0.397–1.929)	0.748	0.292 (0.039–2.206)	0.211
DO	0.563 (0.293–1.081)	0.175	0.400 (0.152–1.055)	0.045	2.063 (0.857–4.963)	0.106
MI	0.650 (0.471–0.897)	0.174	0.667 (0.355–1.251)	0.204	1.333 (0.617–2.883)	0.518

There was a significant correlation between the eae gene carriage and resistance to CN (p = 0.036) in *E. coli*; the entB gene carriage and resistance against DO (p = 0.045) for *K. pneumoniae*; and the pipD gene carriage and resistance to LEV (p=0.039) for *Salmonella *spp. The carriage of the virulence genes was seen to be a stronger risk only for the resistance of *K. pneumoniae* against LEV (OR = 3.230) and CN (OR = 3.000) and *Salmonella *spp. against DO (OR = 2.063).

Association of virulence genes carriage in isolates and risk factors in patients

The risk factors assessed were correlated with virulence gene carriage in both groups of patients and are presented in Table 7. The correlation for the duration of antiretroviral (ARV) treatment was calculated for a duration greater than one year (1) against less than one year (0) of ARV treatment. The correlation for the viral load was calculated for a load higher than (1) against a load lower than (0) the normal for a patient under ARV.

**Table 7:** Association of virulence genes carriage in Escherichia coli, Klebsiella pneumoniae, and Salmonella spp. and risk factors in patients. NA = not applicable, when one variable was either constant or not suited for the given statistical operation (non-binary). LI = lower interval; UI = upper interval; ARV = antiretroviral; TDF = tenofovir disoproxil fumarate; 3TC = lamivudine; EFV = efavirenz; DTG = dolutegravir

Risk factors		*eae* in *E. coli*		*entB* in *K. pneumoniae*		*pipD* in *Salmonella *spp.	
		Odds ratio (LI–UI)	P-value	Odds ratio (LI–UI)	P-value	Odds ratio (LI–UI)	P-value
HIV seropositive		1.872 (0.562–3.218)	0.230	1.563 (0.617–3.959)	0.372	1.942 (0.832–2.646)	0.196
Type of ARV	TDF/3TC/EFV	0.348 (0.199–0.609)	0.076	0.816 (0.382–1.744)	0.622	2.667 (1.090–6.524)	0.009
	TDF/3TC/DTG	0.435 (0.089–2.128)	0.391	1.429 (0.399–5.117)	0.612	0.444 (0.236–0.837)	0.012
Duration of the ARV treatment		1.624 (0.841–2.931)	0.095	1.667 (0.516–5.387)	0.417	0.923 (0.344–2.479)	0.876
ARV therapy		1.327 (0.589–2.989)	0.317	1.563 (0.617–3.959)	0.372	1.833 (0.739–2.546)	0.185
Forget to take ARV		0.783 (0.631–0.971)	0.482	1.204 (0.480–3.024)	0.686	0.778 (0.549–1.103)	0.189
Taking other medical treatment		0.674 (0.548–0.830)	0.078	NA	0.074	2.444 (1.098–3.786)	0.440
Use of antibiotics		NA	NA	1.231 (0.973–1.557)	0.238	1.222 (0.212–2.239)	0.833
Viral load		0.333 (0.108–1.034)	0.073	1.714 (1.513–1.995)	0.031	2.250 (1.137–3.456)	≤0.001

A significant correlation was found between an elevated viral load and the *entB *gene carriage in *K. pneumoniae* (p = 0.031), and between ARV treatment with TDF/3TC/EFV (p = 0.009), ARV treatment with TDF/3TC/DTG (p = 0.012), and an elevated viral load (p = ≤0.001) with the *pipD *gene carriage in *Salmonella* spp. The only factors with a strong risk association with the carriage of virulence genes in isolates were ARV treatment with TDF/3TC/EFV (OR = 2.667), being under medical treatment (OR = 2.444), and an elevated viral load (OR = 2.250) with the pipD gene in *Salmonella *spp.

Association between virulence gene carriage in isolates and severity of symptoms in patients

With the perception of the strength of each symptom being subjective to the patients, the severity of symptoms was estimated as the total number of symptoms associated with the gastroenteritis, with a total of three or more symptoms (1) against less than three (0) correlated with the absence or presence of the virulence gene, as shown in Table 8.

**Table 8:** The relation between the occurrence of virulence genes in isolates and the severity of symptoms in patients.

		*eae* in *E. coli*	*entB* in *K. pneumoniae*	*pipD* in *Salmonella* spp.
HIV-positive	Odds ratio	1.627 (0.756–2.479)	1.833 (0.657–2.983)	2.665 (1.260–4.001)
	P-value	0.017	0.058	0.025
HIV-negative	Odd ratio	1.216 (0.551–2.244)	0.821 (0.487–1.598)	1.645 (1.260–2.901)
	P-value	0.072	0.158	0.042

In HIV-positive patients, there was a significant correlation between the carriage of the *eae *(p = 0.017) and *pipD *(p = 0.025) virulence genes and severe symptoms. A correlation of severe symptoms with virulence gene carriage in HIV-negative patients was found with the *pipD *gene. Although HIV-positive patients with severe symptoms had a higher risk of carrying virulence genes, a strong risk was associated only with the *pipD *gene (OR = 2.665).

## Discussion

To better understand the pathogenicity of strains causing infections, it is necessary to identify their susceptibility to antibiotics and their virulence factors. In this study, we sought to determine the distribution of the virulence genes among *Enterobacteriaceae *isolates from HIV-1 and non-HIV gastroenteritis patients at the Dschang Regional Hospital Annex. We recruited gastroenteritis patients in two groups, namely, one with HIV-positive patients under ARV treatment and the other with HIV-negative patients. From these, we isolated *E. coli*, *K. pneumoniae*, and *Salmonella* spp., which were subjected to antibiotic resistance testing and analysis for the carriage of virulence genes. Correlation tests were done to fine-tune the associations between the various outcomes.

As expected, *E. coli* was the predominant organism isolated in both HIV-positive (30.50%) and HIV-negative patients (31.00%); it is the principal bacterium that normally colonizes the large intestine [18]. This was followed by *K. pneumoniae* (5.00% in HIV-positive and 10.50% in HIV-negative patients), while *Salmonella *spp. was the least prevalent in both HIV-positive (5.50%) and negative patients (7.50%). The fairly similar occurrences of these organisms in HIV-positive and HIV-negative patients can be explained by the fact that these patients were on ARV treatment, which is supposed to boost their immune systems and protect them from opportunistic infections [3].

Results of antimicrobial susceptibility tests revealed that isolates from both HIV-positive and negative patients generally had higher resistance against the beta-lactamase (AMC, CRO, and CTX) and tetracycline (DO and MI) antibiotics. This is because *Enterobacteriaceae *have inherently developed resistance to penicillins, such that association with penicillinase inhibitors has little effect at wild-type minimum inhibition concentrations in standard discs [19]. Moreover, the frequent use of new-generation beta-lactamases (cephalosporins) has been building up resistance against these antibiotics. The high resistance to AMC, DO, and MI means their frequent usage may be a contributing factor in the proliferation of *Enterobacteriaceae *and the development of resistant strains in the gastrointestinal tract [20].

The three virulence genes showed a generally higher occurrence in isolates from HIV-negative patients than HIV-positive patients, with the *eae *gene showing 8.20% against 19.35%, 40.00% against 66.66% for the *entB* gene, and 44.45% against 45.66% for the *pipD *gene. Noting that these genes were isolated across three different *Enterobacteriaceae *species associated with gastroenteritis in patients, it is safe to say that the weakened immune system in HIV-positive patients renders them more susceptible to infections, such that the bacteria need not express virulence genes for extensive colonization and effecting diseases in them. In HIV-negative patients with a strong immune system, infection is controlled by the immune system, such that there is a need for strong expression of virulence genes for extensive colonization and consequently the development of the disease. Several studies have shown that *Enterobacteriaceae *express a plethora of virulence genes to evade the immune response of the host in the face of infection [21,22].

In isolates from HIV-positive patients, resistance to a greater number of antibiotics correlated with virulence gene carriage than in isolates from HIV-negative patients. In HIV-positive patients, *eae *gene carriage correlated with resistance to IPM (p = 0.047) of the beta-lactams, CN (p = 0.007) of the aminoglycosides, and DO (p = 0.029) of the tetracyclines; *entB *gene carriage correlated with resistance to LEV (p = 0.017) of the quinolones; and *pipD *gene carriage correlated with resistance to LEV (p = 0.039) of the quinolones, IPM (p = 0.041) of the beta-lactams, and DO (p = 0.042) of the tetracyclines. DO showed a significant correlation between the three genes. The carriage of the virulence genes was seen to be a stronger risk only for the resistance of *K. pneumoniae* to CRO (OR = 2.286) and CN (OR = 3.000), and *Salmonella *spp. against IMP (OR = 2.750) and DO (OR = 2.118). In HIV-negative patients, the *eae *gene carriage correlated with resistance to CN (p = 0.036) of the aminoglycosides; the *entB *gene carriage correlated with resistance to DO (p = 0.045) of the tetracyclines; and the *pipD *gene carriage correlated with resistance to LEV (p = 0.039) of the quinolones. The carriage of the virulence genes was associated with a stronger risk only for the resistance of *K. pneumoniae* against LEV (OR = 3.230) and CN (OR = 3.000) and *Salmonella *spp. against DO (OR = 2.063). In both HIV-positive and HIV-negative patients, there was a correlation between *eae *gene carriage and resistance to CN and *pipD *gene carriage and resistance to LEV.

These correlations can be explained in the following three main ways: the co-expression of virulence factors and antibiotic resistance; the effect of a strong immune system, which induces higher expression of virulence factors; and the carriage of the genes on plasmids. Monroy-Pérez et al. [23] demonstrated that one-third of the multiple transcription patterns of virulence genes in cervicovaginal *E. coli* strains carried three to seven antibiotic resistance genes. This suggests that the expression of virulence genes goes with that of antibiotic resistance, such that any increase in the expression of virulence genes in isolates will increase their antibiotic resistance. A review by Denzer and Schwerk [24] reported that the survival and intracellular persistence of bacteria during infection depend on the manipulation of innate immune signaling and nutrient supply by means of virulence factors to create a replicative niche. This can especially be true of *Salmonella*, which causes systemic infections. This suggests that a robust immune system in HIV-negative patients would mean more attacks on the bacteria and, as a means of survival, would express these virulence factors, which Monroy-Pérez et al. [23] have shown to be expressed to an extent with antibiotic resistance genes. These virulence genes, for example, *the *eae gene, are borne on plasmids [5], and studies in the same area have shown that antibiotic resistance genes encoding resistance against the tested antibiotics are known to be plasmid-borne, such as the plasmid-mediated extended-spectrum beta-lactamase, quinolone, and aminoglycoside resistance genes [14]. Therefore, there is a possibility of these factors occurring on the same plasmid, being transmitted and expressed together [14]. A review by Cepas and Soto [25] revealed that virulence and resistance are not independent characteristics, but rather there is a relationship between them that depends, among others, on the bacterial species, the specific mechanisms of resistance and virulence, and the immune system of the host. An increase in antibiotic resistance acquired due to selection pressure or from mobile genetic elements enhances virulence capacity.

A significant correlation was found between an elevated viral load and the *entB *gene carriage in *K. pneumoniae* (p = 0.031), and between ARV treatment with TDF/3TC/EFV (p = 0.009), ARV treatment with TDF/3TC/DTG (p = 0.012), and an elevated viral load (p ≤ 0.001) with the *pipD *gene carriage in *Salmonella *spp. The carriage of the *pipD *virulence gene showed a strong risk association with ARV treatment with TDF/3TC/EFV (OR = 2.667), being under medical treatment for another infection (OR = 2.444), and an elevated viral load (OR = 2.250) in *Salmonella *spp. Porco et al. [26] showed that treatment has two possible theoretical effects on the pathogen’s virulence: increasing or decreasing the optimal virulence of a pathogen, depending on host-pathogen interactions. In our study, we observed that medical treatment of gastroenteritis and ARV therapy are both associated with virulence. In Africa, and precisely in Cameroon, most people infected with HIV start ARV treatment at a very late stage of the infection, which would be the cause of an increase in viral load and perhaps several diseases prompting treatment and, by extension, selection pressure on bacteria with virulence genes [27]. The association of resistance with ARV therapy as observed is supported by the findings of a review by Olaru et al. [28], which showed an increased risk of antimicrobial resistance in patients living with HIV across a range of bacterial pathogens, including *E. coli*, and multiple drug classes.

A significant correlation between the carriage of the *eae *(p = 0.017) and *pipD *(p = 0.025) virulence genes and severe symptoms was observed in HIV-positive patients. In HIV-negative patients, the correlation of severe symptoms with virulence gene carriage was found only with the *pipD *gene. Although HIV-positive patients with severe symptoms had a higher risk of carrying virulence genes, a strong risk was associated only with the *pipD *gene (OR = 2.665). These correlations can be explained by the expression of virulence genes leading to the proliferation of the bacteria without a robust countering effect from the depressed immune system of HIV-positive patients, such that they tend to develop several symptoms associated with the disease, including gastroenteritis. This shows that the ability of a microorganism to cause diseases depends not only on their virulence factors but also on the patient’s underlying diseases and other host determinants, a depressed immune system in this case [29].

This study, however, had a few limitations. It treated virulence gene possession and not its expression, and the effect of the gene adopted as the severity of symptoms associated with gastroenteritis could be a far relation given that it is proven by means of probability. Furthermore, the study would have been better if there had been a search for resistance genes responsible for the observed resistance, and the correlation of virulence gene to resistance gene could have been more revealing of their occurrence together in plasmids. These notwithstanding, the findings presented hold their ground as they were founded on robust scientific procedures.

## Conclusions

Antibiotic resistance was associated with virulence gene carriage, an indication that virulence and antibiotic resistance can associate their effects and contribute to poor outcomes in the treatment of bacterial diseases in HIV patients. The possession of virulence genes increased the severity of symptoms associated with gastroenteritis in HIV-positive patients. HIV-negative gastroenteritis patients had a lower occurrence of virulence genes, which permits us to postulate that a robust immune system drives the acquisition of virulence genes as a survival adaptation. The presence of these genes among isolates from both HIV-positive and HIV-negative patients indicates that these genes are in active circulation in our environment.
